# Anti-Amyloid Monoclonal Antibodies for Alzheimer’s Disease: Evidence, ARIA Risk, and Precision Patient Selection

**DOI:** 10.3390/jpm15090437

**Published:** 2025-09-15

**Authors:** Amer E. Alkhalifa, Abdulrahman Al Mokhlf, Hande Ali, Nour F. Al-Ghraiybah, Vasiliki Syropoulou

**Affiliations:** 1 Department of Drug Discovery and Development, Harrison School of Pharmacy, Pharmacy Research Building, Auburn University, Auburn, AL 36849, USA; nfa0007@auburn.edu (N.F.A.-G.); vzs0068@auburn.edu (V.S.); 2 Faculty of Medicine, University of Science and Technology, Sana’a 15034, Yemen; dyn8221706@ju.edu.jo; 3 Faculty of Pharmacy, Zonguldak Bülent Ecevit University, Zonguldak 67100, Türkiye; hande.dos@ef.karaelmas.edu.tr

**Keywords:** Alzheimer’s disease, amyloid-β, monoclonal antibodies, aducanumab, lecanemab, donanemab, amyloid-related imaging abnormalities, APOE ε4, biomarkers, precision medicine

## Abstract

Alzheimer’s disease (AD) is the most common cause of dementia, pathologically defined by extracellular amyloid-β (Aβ) plaques and intracellular tau neurofibrillary tangles. Recent U.S. Food and Drug Administration (FDA) approvals of anti-amyloid monoclonal antibodies (mAbs) aducanumab, lecanemab, and donanemab represent the first disease-modifying therapies for early AD. These therapies have generated both optimism and controversy due to modest efficacy and safety concerns, particularly amyloid-related imaging abnormalities (ARIAs). This review synthesizes current evidence on the efficacy, safety, and biomarker-guided use of anti-Aβ mAbs in AD. **Methods:** We searched PubMed, Scopus, Web of Science, and Google Scholar to 31 July 2025 for studies on anti-amyloid mAbs in AD. Sources included peer-reviewed articles and regulatory reports. The extracted data covered study design, population, amyloid confirmation, dosing, outcomes, biomarkers, ARIA incidence, and management. **Results:** Anti-amyloid mAbs consistently demonstrated robust amyloid clearance and modest slowing of clinical decline in early symptomatic AD. Differences emerged across agents in efficacy signals, safety profiles, and regulatory outcomes. Lecanemab and donanemab showed more consistent cognitive benefits, while aducanumab yielded mixed findings, leading to its withdrawal. ARIAs were the most frequent adverse events, occurring more often in APOE ε4 carriers and typically during early treatment. Biomarker analyses also revealed favorable downstream effects, including reductions in phosphorylated tau and markers of astroglial injury, supporting engagement of disease biology. **Conclusions:** Anti-amyloid mAbs provide proof of concept for AD modification, with the greatest benefit in early disease stages and moderate tau burden. Optimal use requires biomarker confirmation of the amyloid, careful tau staging, and genetic risk assessment. While limitations remain, these therapies represent a pivotal step toward precision neurology and may serve as a foundation for multimodal strategies targeting tau, neuroinflammation, and vascular pathology.

## 1. Introduction

Alzheimer’s disease (AD) is a progressive neurodegenerative disorder clinically marked by insidious memory loss, cognitive decline, and a deterioration in daily functioning [1,2]. AD is the most common cause of dementia, accounting for 60–80% of cases worldwide [3]. As of 2025, 7.2 million Americans aged 65 and older are living with AD, and this number is expected to double to 13 million by 2050 due to the aging U.S. population, where age remains the most significant risk factor [4]. AD disproportionately affects women who represent nearly two-thirds of U.S. cases, as well as older Black and Hispanic Americans, compared to older White individuals [4]. AD is the 10th leading cause of death in the U.S. [4]. Globally, dementia affects approximately 55 million people, with projections estimating that prevalence will double across Europe and nearly triple worldwide by 2050, which highlights the escalating burden of AD as a global public health crisis [4].

Several hypotheses have been proposed to explain the complexity of AD pathology, including the amyloid cascade hypothesis, tau hypothesis, cholinergic hypothesis, oxidative stress theory, and neuroinflammation hypothesis, among others [5]. Despite this diversity, the disease remains pathologically defined by two major hallmarks: extracellular amyloid plaques and intracellular neurofibrillary tangles composed of hyperphosphorylated tau protein [6,7,8,9]. Recent genetic studies and molecular profiling have identified key risk mutations in the amyloid precursor protein (APP), presenilin 1 (PSEN1), and presenilin 2 (PSEN2) genes in early-onset familial AD, and a strong association with the APOE ε4 allele, further reinforcing the amyloid hypothesis and guiding the development of β-amyloid-targeted therapies [10,11,12,13,14,15]. Additional support for the amyloid model comes from the high prevalence of AD in individuals with Down syndrome, who carry an extra copy of the APP gene [16]. The APOE ε4 allele, while strongly associated with late-onset AD [13,14,15], influences disease risk partly by promoting Aβ aggregation and clearance deficits, though it also exerts effects independently of amyloid pathology [17].

Advances in biomarker technologies have confirmed that Aβ accumulation begins years or even decades before clinical symptoms, explaining the long preclinical phase of AD [18]. The amyloid cascade hypothesis, now integrated in the National Institute on Aging–Alzheimer’s Association amyloid/tau/neurodegeneration framework (NIA-AA ATN) biomarker framework, which uses biomarkers of Aβ (A), tau (T), and neurodegeneration (N) to define AD, drives the development of disease-modifying monoclonal antibodies that target upstream Aβ pathology in AD. Accordingly, Aβ pathology is upstream in AD, and consequently, current therapies focus on early intervention, and a slight reduction in amyloid burden might slow or alter disease progression.

Before 2021, all FDA-approved AD drugs were symptomatic therapies. These include acetylcholinesterase inhibitors (donepezil, rivastigmine, galantamine) and the NMDA receptor antagonist memantine [19]. These drugs can modestly improve cognition or behavior in mild-to-moderate AD, but do not change the underlying disease course [20]. In contrast, recent years have seen the first approvals of purported disease-modifying therapies. Aducanumab was the first anti-amyloid antibody to receive regulatory approval for mild AD with confirmed amyloid pathology, followed by lecanemab and donanemab [21]. These antibodies target aggregated Aβ and are indicated for early AD, marking a paradigm shift: they are the first treatments aimed at the disease biology rather than just symptoms [22,23]. These monoclonals are the first AD therapies with true disease-modifying intent. Their benefits (plaque reduction and modest slowing of decline) come at the cost of side effects like amyloid-related imaging abnormalities (ARIAs) and high treatment burden [23].

Given the modest and variable effects of anti-Aβ therapy, it is important to tailor treatment to individual patients. Moreover, as the overall risk–benefit profile for mAbs remains debated, meta-analyses show significantly higher adverse event rates versus a placebo, with amyloid-related imaging abnormalities–edema (ARIA-E) and amyloid-related imaging abnormalities–hemorrhage (ARIA-H) as the most frequent serious adverse reactions [24,25]. Nevertheless, in early AD, anti-amyloid mAbs consistently showed a reduction in amyloid load and a slow Clinical Dementia Rating–Sum of Boxes (CDR-SB) decline by ≈25–30% over 18 months [22,23], which may translate into several months’ delay in the progression of functional decline. Most ARIA events are mild or asymptomatic and resolve with protocolized monitoring and temporary dose suspension, particularly when risk is alleviated by baseline magnetic resonance imaging (MRI), APOE ε4 genotyping, and scheduled follow-up imaging [26,27,28]. Thus, the overall benefit may outweigh the risk in appropriately selected early-stage patients who accept MRI surveillance and shared decision-making, whereas in very high-risk profiles such as ε4/ε4 carriers with microbleeds, risks may outweigh potential benefit [28,29]. Diagnosis and patient selection now rely highly on biomarkers. For example, both lecanemab and donanemab are indicated only for patients with confirmed brain amyloid via PET or CSF according to the ATN model [26,27]. The FDA label for lecanemab even recommends APOE genotyping before treatment to inform ARIA risk. These examples illustrate the shift toward precision treatment, integrating biomarker profiles, genetic risk, and disease stage to guide therapy [29].

This review synthesizes current evidence on the FDA-approved anti-amyloid monoclonal antibodies (mAbs) aducanumab, lecanemab, and donanemab in early symptomatic AD. We integrate (i) mechanisms and epitope selectivity; (ii) efficacy signals across clinical outcomes and biomarkers; and (iii) safety with emphasis on ARIAs, including epidemiology, risk factors, monitoring, and management. Building on this data, we outline a biomarker- and genetics-informed approach to patient selection, treatment initiation, and follow-up. We highlight key gaps and priorities for optimizing real-world use.

## 2. Method

This is a narrative review based on a structured literature search. We performed structured literature searches and synthesized findings narratively, integrating current clinical trial results and regulatory data. We searched PubMed, Scopus, Web of Science Core Collection, and Google Scholar from database inception to 31 July 2025, and consulted regulatory and other gray literature sources relevant to anti-amyloid therapies. Searches were restricted to English-language publications. The strategy combined controlled vocabulary and free-text keywords with Boolean operators: (“Alzheimer* disease” OR AD) AND (amyloid OR Aβ) AND (“monoclonal antibody*” OR aducanumab OR lecanemab OR donanemab) AND (ARIA OR “amyloid-related imaging abnormalit*” OR APOE OR “precision medicine”). Titles and abstracts were screened, followed by full-text review. The data extracted included study design, sample size, population characteristics, amyloid confirmation, dosing regimen, treatment duration, clinical outcomes (CDR-SB, iADRS, ADAS-Cog, ADCS-ADL), amyloid-PET centiloids, fluid biomarkers (p-tau, GFAP), ARIA-E/ARIA-H incidence (overall and stratified by APOE ε4), discontinuation due to ARIAs, and monitoring/management practices.

## 3. The Amyloid Hypothesis

The amyloid hypothesis suggests that abnormal processing of APP leads to the accumulation of Aβ peptides, triggering downstream pathological cascades that contribute to AD onset and progression [30,31]. The hypothesis gained early support from the identification of a pathogenic mutation in the APP gene located on chromosome 21, which was linked to familial early-onset AD and associated with increased deposition of Aβ [32]. This finding contributed to the development of a stepwise model of AD pathogenesis in which Aβ aggregation represents an early event and precedes tau hyperphosphorylation, neurofibrillary tangle (NFT) formation, synaptic dysfunction, and ultimate neuronal death.

Experimental validation of the hypothesis came from transgenic mouse models expressing mutant forms of human APP, such as transgenic mice expressing mutant human APP (PDAPP V717F [APP751 isoform]), which developed Aβ plaques consistent with those observed in human AD brains [33,34]. Furthermore, PET imaging studies have shown that 30% of cognitively normal elderly individuals exhibit cerebral Aβ accumulation, which indicates that Aβ deposition may begin years, if not decades, before the onset of clinical symptoms, thus highlighting its potential role in the preclinical stages of AD [35,36,37,38].

As Aβ is composed of multiple hydrophobic amino acids, the monomer tends to aggregate, forming oligomers, and eventually forming fibrils that stack, forming a plaque [39]. Although still under debate, the exact neurotoxic form of amyloid is yet to be determined, with all sizes, from monomers to fibrils, showing toxicities [40], where amyloid species were associated with increased oxidative stress, reduced blood–brain barrier (BBB) functionality, synaptic toxicities, reduced synaptic plasticity, and neuroinflammation [41,42].

Although the amyloid cascade hypothesis remains the leading model for AD pathogenesis, it is part of an evolving and sometimes controversial debate; accumulating genetic, pathological, and biomarker evidence still strongly supports amyloid as a major initiating event, while recognizing that tau pathology, neuroinflammation, and other mechanisms act in parallel to drive neurodegeneration.

## 4. Monoclonal Antibodies in Alzheimer’s Disease

mAbs revolutionized targeted immunotherapy following the development of hybridoma technology and are now widely used for diagnosis and treatment across multiple diseases [43,44,45,46]. In AD, several mAbs have been developed to target key pathological features, including Aβ, tau aggregates, and neuroinflammatory processes, with several currently under investigation in late-phase clinical trials [47].

**Figure 1:**
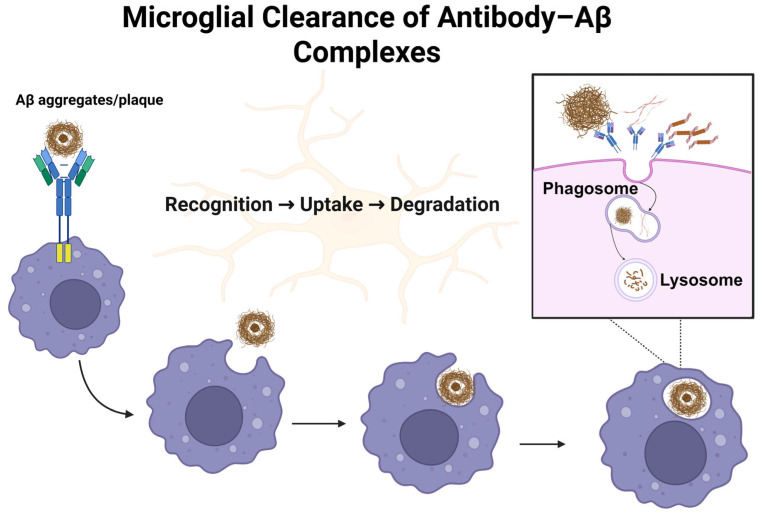
This schematic illustrates the sequential steps of microglial clearance of antibody–Aβ complexes. Antibodies bind and opsonize aggregated Aβ species, enabling their recognition and uptake by microglia. The antibody–Aβ complexes are internalized into phagosomes, which fuse with lysosomes to form phagolysosomes. Within this compartment, enzymatic degradation of the complexes occurs, contributing to the reduction in extracellular Aβ burden.

The disease-modifying therapies in AD have been mainly driven by the amyloid cascade hypothesis, which suggests that the accumulation of Aβ peptides plays a crucial role in the pathological processes. These include tau hyperphosphorylation, synaptic dysfunction, chronic neuroinflammation, and, ultimately, neuronal death, which contribute to progressive cognitive decline and neurodegeneration. Although the amyloid cascade hypothesis has been revised over time and increasingly challenged for its exclusivity in explaining AD pathogenesis, it remains a groundwork for therapeutic development, specifically in the context of immunotherapies targeting Aβ pathology. Among the therapeutic strategies developed to counter Aβ pathology, mAbs have emerged as the most promising disease-modifying approach. Unlike secretase inhibitors or anti-aggregation compounds, mAbs offer a mechanism of action based on passive immunization, enhancing Aβ clearance via microglial activation, phagocytosis, and lysosomal degradation of aggregated Aβ deposits, as shown in Figure 1 [23,48]. These antibodies differ in epitope specificity, with some binding to monomeric Aβ preferentially and targeting toxic oligomeric or fibrillar forms. First-generation mAbs, such as solanezumab, crenezumab, and gantenerumab, largely failed in clinical trials due to limited efficacy in cognitive endpoints and insufficient plaque clearance [49,50,51]. Notably, solanezumab was unable to engage its target effectively in multiple large trials spanning the preclinical to moderate stages of AD, reinforcing the idea that targeting monomeric Aβ alone may be insufficient for altering disease progression [49,52].

These early failures prompted a shift toward developing second-generation mAbs designed to recognize and neutralize aggregated Aβ species such as protofibrils, fibrils, and dense-core plaques [23]. This new generation of anti-amyloid mAbs, including aducanumab, lecanemab, and donanemab, demonstrated marked reductions in amyloid plaque burden as visualized by amyloid PET imaging, as well as statistically significant, albeit modest, slowing of cognitive decline in early-stage AD populations [53,54,55,56].

Despite these milestones, the field continues to face critical challenges. The clinical efficacy of these agents remains limited, with most studies reporting only modest cognitive benefits. Moreover, these treatments are associated with potentially serious adverse events, particularly ARIAs, which occur more frequently in patients with the APOE ε4 genotype carriers [56]. The commercial viability of some antibodies has also been questioned; aducanumab, for example, was withdrawn from the market in 2024 because of limited uptake and ongoing concerns over benefit–risk balance [55].

Nevertheless, the development of mAbs for AD has marked a turning point in therapeutic strategy. After more than two decades of clinical trials aimed at β-amyloid clearance, including early attempts at active vaccination and secretase inhibition that failed due to safety or efficacy issues, passive immunization with humanized mAbs has emerged as the most viable pathway. These antibodies differ not only in their epitope specificity and pharmacokinetic properties but also in their clinical efficacy and safety profiles and the risk mitigation strategies employed collectively, offering a foundation for patient stratification and the advancement of personalized therapeutic approaches [57]. In particular, the reliance on biomarker confirmation of amyloid pathology (PET or CSF Aβ levels) before treatment initiation reflects a growing movement toward precision medicine in AD [58].

### 4.1. Aducanumab (Aduhelm^®^)

**Figure 2:**
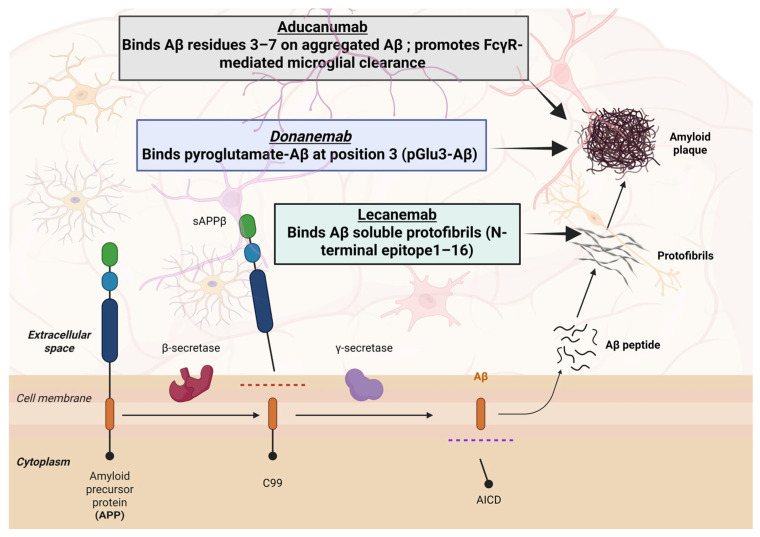
This schematic illustrates the amyloidogenic pathway and antibody binding sites. Sequential cleavage of APP by β- and γ-secretases produces Aβ peptides, which aggregate into protofibrils and plaques. Aducanumab targets aggregated Aβ residues 3–7, donanemab binds pyroglutamate-Aβ at position 3 (pGlu3–Aβ), and lecanemab recognizes soluble Aβ protofibrils at the N-terminal epitope (1–16).

Aducanumab is a fully human IgG1 monoclonal antibody originally derived from aged individuals exhibiting minimal cognitive decline [59]. This antibody shows high selectivity for Aβ aggregated forms, including fibrils and soluble oligomers, while demonstrating minimal affinity for monomeric Aβ species [59,60], as shown in Figure 2. It was granted accelerated approval by the U.S. FDA on 7 June 2021 [59], becoming the first therapy approved to modify the underlying pathology of AD and the first new treatment for AD since 2003 [61,62]. Developed jointly by Biogen and Eisai, its approval was granted based on its demonstrated ability to reduce amyloid plaque burden, as measured by PET imaging, rather than conclusive evidence of clinical efficacy [61,63]. It is administered via intravenous infusion every 4 weeks, with an initial dose titration starting at 1 mg/kg, increasing to a maintenance dose of 10 mg/kg as tolerated [64]. While this decision provides a critical regulatory milestone, it has also gained widespread controversy across scientific, medical, and policy domains, raising essential questions about the criteria for accelerated approval in neurodegenerative diseases. The European Medicines Agency and Japan’s PMDA refused marketing authorization for aducanumab [65]. Biogen later announced the discontinuation of aducanumab in 2024, ending commercialization by November 2024 [66].

#### Clinical Development and Efficacy

In the Phase 1b PRIME clinical study, 165 participants were enrolled; these participants were diagnosed with early symptomatic AD characterized by either mild cognitive impairment (MCI) or mild dementia, and confirmed amyloid positivity via PET imaging [60]. Aducanumab demonstrated a dose-dependent reduction in cerebral amyloid burden, with high-dose treatment nearly normalizing amyloid PET signals to levels observed in non-AD individuals. However, the ARIAs, particularly ARIA-E (edema), were also dose-dependent. ARIA-E occurred in up to 41% of participants at the highest dose, particularly among APOE ε4 carriers [60]. Most ARIA cases were asymptomatic, although some patients experienced headaches, visual disturbances, or confusion. Approximately 44% of patients who developed ARIA-E discontinued treatment.

Two pivotal Phase 3 trials, EMERGE and ENGAGE, were initiated to assess clinical efficacy in early AD [67]. Both trials initially appeared futile based on interim analyses, prompting Biogen to halt the studies in March 2019. However, subsequent post hoc analyses indicated that the high-dose arm of EMERGE met its primary endpoint, showing a 22% relative reduction in clinical decline as measured by the CDR–Sum of Boxes (CDR-SB), while ENGAGE failed to replicate these results [67].

The ICARE AD post-marketing study (NCT05097131) was terminated in May 2022 due to low enrollment, with only 29 participants enrolled [66]. Scrutiny of the approval process intensified, culminating in a congressional investigation that criticized both the FDA and Biogen for procedural irregularities and excessive collaboration [68]. The Institute for Clinical and Economic Review (ICER) estimated a cost-effective price between USD 3000 and USD 8400 per year, far below Biogen’s launch price of USD 56,000 [69].

The rise and fall of aducanumab, from its historic status as the first monoclonal antibody approved by the FDA for AD to its eventual market withdrawal, highlights the profound challenges and intricate complexities that continue to impede the development of effective therapeutics for AD. While it confirmed the feasibility of amyloid clearance in humans, its modest clinical efficacy and the regulatory controversy surrounding its approval underscore the urgent need for more rigorous and clearly defined clinical benefit, safety, and patient selection standards to advance future AD therapeutics. Significantly, its experience influenced the coverage of subsequent amyloid-targeting antibodies such as lecanemab and donanemab, which demonstrated both target engagement and statistically significant cognitive benefit in trials. These agents received broader Centers for Medicare & Medicaid Services (CMS) support under the NCD with coverage of 80% contingent on enrollment in CMS-approved registries. Aducanumab thus represents a pivotal case in the evolving landscape of AD treatment policy and drug development.

### 4.2. Lecanemab (Leqembi^®^)

Lecanemab is a humanized IgG1 monoclonal antibody engineered from the murine mAb158 and designed to selectively target the intermediate aggregates of soluble Aβ protofibrils implicated in synaptic dysfunction and neurotoxicity in AD [70]. By binding these protofibrils, lecanemab facilitates their clearance from the brain, which in turn leads to a secondary reduction in insoluble amyloid plaque deposition [71]. Lecanemab is administered intravenously biweekly at a dose of 10 mg/kg, without the requirement for dose titration [71]. In January 2023, lecanemab was granted accelerated approval from the U.S. FDA, followed by full approval in July 2023, for the treatment of patients with mild cognitive impairment or mild Alzheimer’s dementia, depending on confirmation of brain amyloid pathology through PET imaging or cerebrospinal fluid biomarkers [71].

#### Clinical Development and Efficacy

Preclinical investigations using the Tg-ArcSwe mouse model, which expresses human APP mutations associated with familial AD, demonstrated that both short- and long-term administration of mAb158 reduced Aβ protofibril levels in brain tissue and cerebrospinal fluid (CSF), supporting the antibody’s potential to modify disease pathology [72]. Lecanemab was first evaluated in humans through a Phase 1, single- and multiple-ascending dose study (NCT01230853), involving 80 patients with mild-to-moderate AD [73]. The trial confirmed the drug’s safety and tolerability across escalating doses. Serious adverse events (SAEs) were rare, and the incidence of ARIAs, including ARIA-E and ARIA-H (microhemorrhages), was comparable to a placebo [73].

In the subsequent Phase 2 trial (NCT01767311), 856 participants with MCI or mild AD and biomarker-confirmed amyloid pathology were enrolled [74]. Patients receiving biweekly doses of 10 mg/kg lecanemab showed meaningful clinical responses over 18 months. Although the primary Bayesian analysis at 12 months did not meet its pre-specified threshold (64% probability of achieving >25% improvement in AD Composite Score (ADCOMS) vs. 80% target), secondary outcomes demonstrated significant cognitive benefit at 18 months: a 30% improvement in ADCOMS, 47% in 14-item AD Assessment Scale—Cognitive subscale (ADAS-Cog14), and 26% in CDR-SB, compared to a placebo. Biomarker analysis also revealed reductions in brain amyloid load and CSF phosphorylated tau (p-tau) levels. The incidence of ARIA-E in this high-dose group was 9.9%, rising to 14.3% among APOE ε4 carriers [74]. A disease simulation model based on data from this study suggested that early and sustained administration of lecanemab could significantly delay disease progression, supporting its use in early-stage AD [75].

The pivotal Phase 3 CLARITY AD trial (NCT03887455) enrolled 1906 participants with MCI or mild dementia due to AD and confirmed amyloid positivity [66]. Over 18 months, lecanemab slowed cognitive decline by 27% on the primary endpoint in CDR-SB compared to a placebo. Secondary endpoints also favored lecanemab, including a 26% improvement in ADAS-Cog14, 24% in ADCOMS, and 37% in ADCS-ADL-MCI. Additionally, lecanemab reduced the amyloid PET signal by 59.1 centiloids (from 77.9 to 18.8 CL), indicating substantial plaque clearance. ARIA-E and ARIA-H occurred in 12.6% and 17.3% of lecanemab-treated participants, respectively, with symptomatic events in 2.8% (ARIA-E) and 0.7% (ARIA-H) [66,76,77]. Based on these results, the FDA granted accelerated approval to lecanemab in January 2023, followed by full traditional approval in July 2023 [78]. Under the Centers for CMS and the NCD policy for amyloid-targeting monoclonal antibodies, lecanemab became eligible for broader coverage. CMS reimburses 80% of treatment costs contingent on enrollment in a CMS-approved registry. These developments positioned lecanemab as one of the first anti-Aβ therapies to demonstrate both biomarker engagement and cognitive efficacy in a large-scale clinical setting [79,80].

### 4.3. Donanemab (Kisunla^®^)

Donanemab is a humanized IgG1 monoclonal antibody developed by Eli Lilly for early AD [80]. Initial United States approval of donanemab came in 2024 for use in patients with mild cognitive impairment or mild dementia due to Alzheimer’s with confirmed amyloid pathology. The recommended dosing regimen is 700 mg given intravenously every four weeks for the first three infusions, then 1400 mg IV every four weeks thereafter [81].

Donanemab’s activity is highly plaque-specific [82]. It binds to the pyroglutamate-modified Aβ epitope (N3pE) present only on fibrillar, plaque-associated Aβ [83]. This binding effectively “tags” existing amyloid plaques and triggers microglia to engulf and clear these fibrillar Aβ aggregates [54,83]. Because soluble, monomeric Aβ does not contain the N3pE modification, donanemab largely spares non-plaque Aβ species in the brain [82,83].

The primary safety concern with donanemab, as with similar anti-amyloid antibodies, is the occurrence of ARIAs [83]. In clinical trials of donanemab, ARIA-E (vasogenic cerebral edema or effusion) was observed in roughly 24% of patients receiving the drug (about 6% of those patients experienced symptomatic edema), compared to only around 2% of patients receiving a placebo [83]. ARIA-H, which can manifest as small microbleeds or superficial siderosis, occurred in approximately 31% of donanemab-treated patients versus about 13% in the placebo group. Other common adverse effects reported in 10% or more of patients included headache and infusion-related reactions [83]. Patients who are APOE ε4 homozygotes have been found to have an exceptionally high risk of ARIAs with therapies like donanemab [83]. To date, there have been no indications of significant liver toxicity or other end-organ damage attributable to donanemab in trials [83].

In preclinical studies using transgenic mouse models of Alzheimer’s (PDAPP mice), a murine precursor of donanemab was shown to reduce amyloid plaque burden in a dose-dependent manner without causing microhemorrhages [82].

#### Clinical Development and Efficacy

In early clinical studies (Phase I trials NCT01837641 and NCT02624778), a total of 161 patients with mild cognitive impairment or mild-to-moderate AD received donanemab in single-ascending dose and multiple-ascending dose experiments, with doses up to 10–40 mg/kg administered intravenously [84,85]. These Phase I trials demonstrated that donanemab could substantially reduce amyloid plaque levels even at relatively low doses: for instance, monthly infusions of 10 mg/kg led to approximately a 40–50% reduction in brain amyloid plaque as measured by amyloid PET imaging [84,85].

A subsequent Phase II trial (TRAILBLAZER-ALZ, NCT03367403) was conducted over 76 weeks in 272 patients with early symptomatic AD [86]. All participants in this study were confirmed to have brain amyloid pathology by PET and had an intermediate level of tau pathology (a criterion for enrollment). In this trial, patients were randomized to receive donanemab (at the dosing schedule later used in Phase III) or a placebo. Donanemab treatment led to a statistically significant slowing of clinical decline compared to a placebo on the primary outcome measure, the Integrated AD Rating Scale (iADRS), which combines cognitive and functional assessments. Over 76 weeks, patients on donanemab declined by 6.86 points on the iADRS, whereas those on a placebo declined by 10.06 points, yielding a between-group difference of +3.20 points (95% confidence interval 0.12 to 6.27; *p* = 0.04). While not all secondary endpoints reached statistical significance, most cognition and daily functioning measures showed numerical trends favoring donanemab over a placebo.

Biomarker findings from TRAILBLAZER-ALZ were striking: donanemab produced a reduction in brain amyloid load that was about 85 centiloids greater than that seen in the placebo group, and approximately 68% of patients in the donanemab arm converted to an amyloid-negative status on PET by the end of the study (defined as an amyloid burden below 24.1 centiloids). There was minimal difference between groups in the change in the global tau PET signal (~0.01 centiloid difference), suggesting that tau pathology did not significantly worsen relative to a placebo during the 76 weeks. Additionally, exploratory blood biomarkers indicated potential disease-modifying effects: plasma phosphorylated tau (p-tau_217) levels decreased by about 23% from baseline in the donanemab-treated group (whereas they tend to increase in progressing AD), and levels of glial fibrillary acidic protein (GFAP), an astrocytic injury marker, fell by roughly 12%. In terms of safety, the Phase II trial’s ARIA incidence was consistent with Phase I observations: symptomatic ARIA-E occurred in 6.1% of patients on donanemab (with virtually no cases in the placebo group), and there were no reported amyloid-related deaths or seizures. The overall positive findings from this Phase II study provided a strong rationale for proceeding to a larger Phase III trial to confirm efficacy and safety [86].

The confirmatory Phase III trial, TRAILBLAZER-ALZ 2 (NCT04437511), enrolled approximately 1736 patients with early AD, including both individuals with intermediate tau levels and those with high tau pathology (around 68% of participants had low or medium tau and 32% had high tau, stratified to ensure representation of both subgroups) [87]. Participants were treated with donanemab (700 mg for the first three doses, then 1400 mg IV every four weeks) or a placebo for 18 months.

Donanemab, consistent with previous outcomes, demonstrated a significant slowing of clinical decline relative to a placebo. Among the low/medium tau subgroup, the primary population for analysis, the average change on the iADRS from baseline to 76 weeks was—6.02 points in the donanemab group versus—9.27 in the placebo group. This represents a difference of +3.25 points (95% CI 1.88–4.62; *p* < 0.001) favoring donanemab. Similarly, on the CDR-SB, patients receiving donanemab worsened by 1.20 points on average, compared to a 1.88-point worsening in the placebo group, a difference of—0.67 (with *p* < 0.001). These treatment effects correspond to approximately a 35% relative slowing of decline on the CDR-SB and around a 40% slowing on the iADRS for the donanemab group compared to a placebo over the 18 months [87]. Donanemab’s impact on amyloid plaques in Phase III was profound: the mean amyloid PET centiloid value dropped by roughly 88 points from baseline in the donanemab-treated patients, whereas it remained essentially unchanged in those on a placebo. By the end of the trial, about 80% of patients treated with donanemab had achieved complete clearance of amyloid plaques on their PET scans. Consistent with this, plasma biomarkers showed robust responses; for example, plasma p-tau_217 levels fell by approximately 39% from baseline in the donanemab group (again indicative of a potential slowing of Alzheimer’s pathological progression), and GFAP levels were reduced by about 21%.

The safety findings in Phase III mirrored those observed earlier. ARIA-E was detected in 24% of patients on donanemab (around 6% experienced symptoms from the edema) compared to a much lower incidence in the placebo group. ARIA-H was seen in about 31% of donanemab patients versus 13% of placebo patients. Infusion-related reactions occurred in 8.7% of individuals receiving donanemab compared to 0.5% of those on a placebo. There were three deaths among donanemab-treated patients that were considered possibly related to treatment (such as complications from ARIAs), whereas one patient in the placebo group had a death considered to be possibly treatment-related. In total, donanemab met 23 out of 24 prespecified endpoints in the TRAILBLAZER-ALZ 2 study, a result that strongly confirmed its clinical efficacy and supported its benefit–risk profile, leading to expectations of regulatory approval (see Table 1).

In addition to the completed trials, several studies are ongoing to further characterize donanemab’s efficacy and optimal use. TRAILBLAZER-ALZ 3 is a large Phase III prevention trial enrolling roughly 3300 cognitively normal individuals who have evidence of brain amyloid accumulation (as determined by PET scans) [88]. The goal of this study is to evaluate whether intervening with donanemab at this preclinical stage can delay the development of symptomatic AD; the primary outcome is the time to progression as measured by the CDR scale [88].

TRAILBLAZER-ALZ 4 was an open-label, head-to-head clinical trial designed to compare the amyloid plaque-clearing efficacy of donanemab versus aducanumab, both anti-amyloid monoclonal antibodies, in approximately 200 participants with early symptomatic AD. The primary endpoint was the extent and rate of amyloid clearance as measured by PET imaging. The results demonstrated that donanemab achieved significantly greater amyloid plaque reduction at 6 months, with a notably higher proportion of participants reaching amyloid negativity compared to those receiving aducanumab [89].

Furthermore, TRAILBLAZER-ALZ 5 (target enrollment around 1500) and TRAILBLAZER-ALZ 6 (around 800 participants) are additional Phase III trials planned to investigate long-term outcomes with donanemab, explore alternative dosing regimens (for example, determine whether less frequent dosing could maintain benefits after plaque clearance), and refine monitoring protocols and management strategies for ARIAs and other adverse events during treatment [90,91].

From a regulatory standpoint, the path to approval for donanemab has involved careful consideration of its safety data. After the completion of the Phase II TRAILBLAZER-ALZ trial, Eli Lilly initially sought accelerated approval for donanemab [91]. However, in that first review cycle, the U.S. FDA issued a complete response letter, citing the need for more extensive safety data, specifically noting that fewer than 100 patients had been exposed to donanemab for at least one full year at the time, which was considered insufficient to fully assess long-term safety. In response, Lilly continued with the Phase III program, and the robust results from TRAILBLAZER-ALZ 2 have since provided a much larger dataset supporting both the drug’s efficacy and the manageability of its risks [91]. Consequently, Lilly has submitted a new application seeking traditional (full) approval for donanemab in the United States. Similar regulatory submissions are underway in the European Union. The expectation is that the comprehensive Phase III findings will satisfy regulators regarding donanemab’s clinical benefit and safety profile, potentially leading to approval and availability of this therapy for patients with early AD.

**Table 1:** Summary of selected clinical trials of anti-amyloid mAbs in AD.

**NCT Number**	**Phase**	**Design Details**	**Results and References**
**Aducanumab**			
NCT05108922	Phase 3	Open-Label, Parallel-Group, 2-Arm Study.	At 6 months, 37.9% of patients achieved amyloid clearance compared with 1.6% in the aducanumab group. The median time to clearance was shorter with donanemab (359 days) than with aducanumab (568 days). In addition, donanemab produced a greater reduction in phospho-tau at 6 months, although this difference diminished with continued treatment [92].
NCT02484547	Phase 3	Randomized, Parallel assessment, Double-Blind.	High-dose treatment produced a statistically significant reduction in decline across CDR-SB, ADAS-Cog13, ADCS-ADL-MCI, and MMSE, whereas the low-dose group showed no significant difference from placebo [93].
NCT02477800	Phase 3	Randomized, Parallel assessment, Double-Blind.	Neither high- nor low-dose treatment groups showed significant cognitive benefit over placebo across assessed endpoints [93].
NCT05310071	Phase 3	Randomized, Parallel Assignment, quadruple masking (patient, care provider, PI, outcome assessing researcher).	Specific statistical analysis results not published [94].
**Donanemab**			
NCT05108922	Phase 3	Open-Label, Parallel-Group, 2-Arm Study.	At 6 months, 37.9% of patients achieved amyloid clearance versus 1.6% with aducanumab. Median clearance time was shorter with donanemab (359 days) than with aducanumab (568 days). Donanemab also showed greater phospho-tau reduction at 6 months, though this effect decreased with continued treatment [89].
NCT04640077	Phase 2	Non-randomized, sequential.	The donanemab reduction of tau was more pronounced in patients with complete amyloid clearance [95].
NCT04437511	Phase 3	Randomized, parallel, double masking (patient, investigator).	Cognitive decline was slow compared to the placebo group. Amyloid clearance significantly increased, with no change in tau pathology [87].
NCT03367403	Phase 2	Randomized, parallel, double masking (patient, investigator).	Significant slowing of clinical decline (in iADRS and ADAS, no significant difference in CDR-SB and MMSE), increase in amyloid clearance, mixed tau pathology outcome [86].
**Lecanemab**			
NCT03887455	Phase 3	Randomized, parallel, quadruple masking (patient, care provider, PI, outcome assessing researcher).	At 18 mo, slowed decline (CDR-SB −27%, ADAS-Cog 14–26%, ADCOMS −24%, ADCS-ADL-MCI −37% vs. placebo); reduced brain amyloid by 59 CL; ARIA-E 12.6% (symptomatic 2.8%), ARIA-H 17.3% (symptomatic 0.7%); led to FDA traditional approval [96].
NCT01767311	Phase 2	Randomized, parallel, triple masking (patient, care provider, PI).	Did not meet 12 mo primary endpoint; at 18 mo slowed decline (ADCOMS −30%, ADAS-Cog 14–47%, CDR-SB −26% vs. placebo); reduced brain Aβ and CSF p-tau; ARIA-E 9.9% (14.3% in APOE ε4) [97].
NCT01230853	Phase 1	Randomized, quadruple masking (patient, care provider, PI, outcome assessing researcher).	This study showed that lecanemab was safe and well-tolerated across all treatment groups, even at progressively increasing doses [98].

### 4.4. Cognitive Outcome Measures and Clinical Meaningfulness

Clinical trials for AD rely on standardized cognitive and functional scales to measure the therapeutic effects; however, the relationship between these numeric changes and meaningful improvements in daily life is complex. Common endpoints include the CDR-SB, which assesses six domains of cognition and function (score range 0–18, the higher is the worse) [99,100]; the Alzheimer’s Disease Assessment Scale–Cognitive Subscale (ADAS-Cog, 11- or 14-item versions), which assesses memory and other cognitive functions (higher scores indicate worse cognition); and composite indices like the integrated AD Rating Scale (iADRS), which combines ADAS-Cog and functional measures (ADCS-iADL) into a single score [99].

In clinical practice, simpler tests (MMSE, MoCA) and functional questionnaires are often used, but they align broadly with the domains captured in trials [100]. Defining a “meaningful” change on these scales remains challenging. Clinicians and regulators consider a change that reaches the minimal clinically important difference (MCID) as potentially noticeable to patients or caregivers [99]. For early AD, an MCID has been estimated as being around 1–2 points on the CDR-SB and ~2–4 points on ADAS-Cog [100]. Within-patient CDR-SB increases of ≥1–2 points over 12–18 months are associated with detectable worsening in global clinical status [100]. Notably, however, the treatment effects observed with anti-amyloid antibodies fall well below these thresholds. For example, in the Phase 3 CLARITY-AD trial of lecanemab, the drug group declined 0.45 points less on CDR-SB than the placebo over 18 months (1.21 vs. 1.66)—a 27% slowing of decline that was statistically significant [99]. On ADAS-Cog14, the difference was 1.44 points (4.14 vs. 5.58) [101]. Such differences, while statistically positive, are small in absolute terms [25]. Indeed, a recent meta-analysis confirmed that none of the cognitive or functional changes with aducanumab, lecanemab, or donanemab exceeded the accepted MCID [25]. This means that the average patient might not perceive a clear improvement or stabilization from these drugs over 18 months—the benefit manifests as a modest slowing of decline on tests, rather than dramatic recovery of lost abilities [102].

In practical terms, a ~25% slower decline may translate to only a few months’ delay in the progression of symptoms, which may be imperceptible in day-to-day functioning over a year or two [76]. There is an ongoing debate about whether such statistically significant changes correspond to “tangible” benefits for patients in daily life [102]. Patient-centered outcomes like quality of life (QoL) and functional independence are crucial when interpreting these effects. Early in AD, patients often maintain relatively good QoL and compensate for mild cognitive deficits [103,104]. A small slowing of cognitive decline, a 0.5-point CDR-SB difference for example, is unlikely to noticeably improve the ability to manage daily tasks or reduce caregiver burden in the short term [102]. Moreover, QoL is influenced by many factors outside cognition, mood, social support, and physical health; so a slight cognitive benefit may not substantially change [103,104,105].

This helps explain why a ~30% slowing in decline on paper may not feel “worth it” to some patients. Longer-term studies are still needed to determine whether early slowing of cognitive decline translates into meaningful preservation of function, such as delaying loss of independence. Encouragingly, recent long-term data are beginning to address this gap. Over three years of treatment, including both the core study and open-label extension (OLE), lecanemab reduced cognitive decline on CDR-SB by 1.01 points compared to the expected decline observed in the ADNI cohort, and by 1.40 points when benchmarked against the BioFINDER cohort [106]. This benefit grew more pronounced after four years, with a reduction of 1.75 points versus ADNI and 2.17 points versus BioFINDER, roughly tripling the effect seen in the original 18-month trial period. Similarly, donanemab’s benefit roughly doubled after three years of continuous therapy [107]. These findings, presented at the 2025 Alzheimer’s Association International Conference (AAIC), highlight the potential for sustained treatment to yield greater clinical benefit over time and support the case for early initiation and adherence to therapy [106].

## 5. Amyloid-Related Imaging Abnormalities (ARIAs)

### 5.1. Pathophysiology of ARIAs

An ARIA is primarily detected through MRI; yet its exact pathophysiology remains incompletely understood. It is thought to be a result of mAbs binding to Aβ deposits in the vasculature and brain parenchyma, which leads to plaque mobilization and subsequent clearance [108]. This process can transiently lead to BBB dysfunction, causing extravasation of fluid (ARIA-E) or blood-derived products (ARIA-H) [109]. Because Aβ is involved in both AD and cerebral amyloid angiopathy (CAA), which co-occur in up to 80% of patients, the site of Aβ deposition (vascular in CAA vs. parenchymal in AD) demonstrates several of their clinical differences [110,111]. CAA is characterized by Aβ deposition in cortical and leptomeningeal vessel walls, resulting in vessel fragility, microhemorrhages, and impaired perivascular clearance. Targeting and clearance of amyloid by mAbs may transiently increase vascular amyloid load and provoke perivascular inflammation, thus exacerbating ARIAs such as microhemorrhages, superficial siderosis, or vasogenic edema [110,111,112,113].

**Figure 3:**
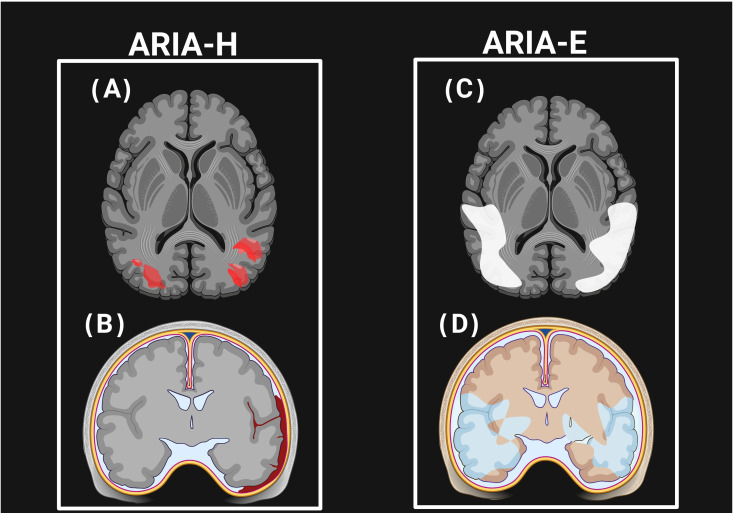
This schematic illustrates amyloid-related imaging abnormalities (ARIAs). ARIA-H (**A**,**B**) is represented by multiple cerebral microhemorrhages (small red-brown foci at the gray–white junction) and superficial siderosis (thin cortical rim), typically visualized as hypointense lesions on T2/SWI MRI. ARIA-E (**C**,**D**) is shown as patchy white matter hyperintensities in parietal–occipital and periventricular regions, consistent with vasogenic edema or effusion detected on FLAIR MRI. These patterns highlight the radiological hallmarks of ARIAs observed in patients treated with anti-amyloid monoclonal antibodies.

MRI features of ARIA-H closely mimic those of CAA, while ARIA-E shares imaging features with inflammatory CAA [112,113]. Therefore, CAA and its inflammatory variants provide a useful model for understanding ARIA pathophysiology and risk stratification [108], as shown in Figure 3.

Current debates focus on whether the presence of CAA should affect patient selection and treatment decisions for anti-amyloid mAbs. Some experts caution that removing a vascular amyloid may destabilize fragile vessels, increasing the risk of hemorrhage and ARIAs, particularly in patients with MRI markers of CAA such as lobar microbleeds or superficial cortical siderosis. Conversely, emerging evidence suggests that untreated CAA contributes to progressive cognitive decline and may accelerate the disease course [114]. This duality has prompted calls for improved biomarkers, such as imaging of perivascular drainage, to guide safer patient selection and therapy development [115]. The clinical implications are significant. Pre-treatment susceptibility-weighted MRI screening is recommended to identify patients with extensive microhemorrhages or siderosis, who may require more cautious dosing or may be excluded from therapy. Future research should explore individualized treatment strategies, such as lower starting doses or modified titration schedules, to minimize ARIA risk in patients with severe CAA while preserving therapeutic efficacy.

### 5.2. Incidence of ARIA-E and ARIA-H

Since the early 2000s, several clinical trials have evaluated mAbs targeting Aβ in AD, many reporting ARIAs as a major safety concern [109,116,117]. First-generation mAbs, such as bapineuzumab, solanezumab, gantenerumab, crenezumab, and ponezumab, were the first to be evaluated in large-scale studies [118]. Bapineuzumab, a humanized IgG1 antibody targeting the N-terminus of Aβ, was the first agent introduced in humans [116,119]. APOE ε4 carriers had significantly higher ARIA-E incidence (15.3%) compared to noncarriers (4.2%) [119], with most cases occurring after the first infusion and recurring in 25.6% upon retreatment [109,120]. Because of the high rate of symptomatic ARIA-E at doses >2 mg/kg, the drug’s development was stopped early [119]. ARIA-H, while not dose-dependent, was associated with baseline microhemorrhages and concurrent antithrombotic use [109].

Solanezumab, which binds Aβ_16–26_, demonstrated a low ARIA-E incidence (0.9%) and ARIA-H incidence (4.9%) in the combined Phase 3 EXPEDITION trials that included over 2000 participants [121]. Cognitive endpoints such as MMSE and CDR-SB were met in EXPEDITION 3, though the primary endpoint (ADAS-Cog14) was not [121]. Gantenerumab, which binds insoluble fibrils at conformational epitopes, was tested in the Scarlet Road study using doses up to 225 mg [116]. APOE ε4 carriers experienced higher rates of both ARIA-E (15%) and ARIA-H (19.4%) compared to noncarriers (11% and 11%, respectively), with more than 80% of ARIA-E cases asymptomatic [116].

Crenezumab, a humanized IgG4 antibody designed to reduce Fc receptor-mediated microglial activation, demonstrated no ARIA-E and low incidence of ARIA-H (4.9%) at doses up to 60 mg/kg, and no ARIA at 120 mg/kg [116]. Ponezumab, an IgG2 antibody targeting Aβ40’s C-terminus, showed very low ARIA-E (0.7%) but a relatively high ARIA-H incidence (16.4%) [122]. However, due to limited clinical efficacy, both crenezumab and ponezumab were discontinued [109,116].

In contrast, the second generation of mAbs therapies, such as lecanemab, donanemab, and aducanumab, demonstrated stronger clinical efficacy alongside increased ARIA rates, particularly in APOE ε4 carriers [48]. Lecanemab was studied in a Phase 2b study of 854 patients and showed ARIA-E in 14.3% of APOE ε4 carriers and 8.0% of noncarriers; ARIA-H was reported in 13.1% and 4.6%, respectively [74,109,123].

Donanemab, evaluated in the TRAILBLAZER-ALZ Phase 2 trial, showed a comparatively high incidence of ARIA-E (27.5%) and ARIA-H (30.5%) [109]. Aducanumab was studied in the EMERGE and ENGAGE Phase 3 trials [124]. In EMERGE, ARIA-E occurred in 2%, 26%, and 35% of patients in the placebo, low-dose, and high-dose groups, respectively, while ENGAGE reported similar rates (3%, 26%, 36%) [67]. APOE ε4 carriers in the high-dose arms had markedly higher ARIA-E rates (43% vs. 18% in EMERGE; 42% vs. 23% in ENGAGE) [67]. ARIA-H also occurred more frequently in patients who developed ARIA-E, particularly in the form of microhemorrhages and superficial siderosis [67].

A notable case report documented severe ARIA-E and ARIA-H in a 66-year-old APOE ε4 carrier undergoing aducanumab dose escalation, who presented with encephalopathy, alexia, and malignant hypertension, requiring ICU admission and corticosteroid therapy [125]. While ARIA-E resolved over six months, ARIA-H persisted. The FDA label for aducanumab includes detailed guidance on administration, monitoring, and ARIA management [126].

### 5.3. Risk Factors Associated with ARIAs

Numerous risk factors for ARIAs have been recognized, with APOE ε4 carriers being the most significant for both ARIA-E and ARIA-H [60,109,127,128,129]. Typically, APOE ε4 carriers develop a higher Aβ burden in brain tissue and vasculature, which may lead to impaired perivascular clearance, increased vascular permeability, and subsequent development of ARIA-E or ARIA-H [109,130].

ARIA-E shows a dose-dependent relationship, occurring more frequently at higher doses of anti-Aβ antibodies. In high-dose aducanumab trials, ARIA-E was observed in up to 55% of APOE ε4 carriers [109]. ARIA-E usually occurs in the early stages of the treatment, often with symptoms, but typically resolves after stopping therapy. Recurrence after dose reinitiation has been reported in 13.8–25.6% of cases [56].

ARIA-H is often asymptomatic and may be identified incidentally via MRI [131]. Use of antithrombotic medications increases the risk of ARIA-H, especially in patients with pre-existing microhemorrhages [132,133]. The presence of ≥5 microhemorrhages or any superficial siderosis has been proposed as an exclusion criterion for anti-Aβ therapy trials, though not consistently applied [109].

Cardiovascular comorbidities such as hypertension, diabetes, and hyperlipidemia have been investigated, but to date, no statistical relationship with ARIA risk has been reported [134]. Given the elevated ARIA rates in homozygous APOE ε4 carriers, genetic testing may be advisable before starting anti-Aβ therapy [109].

### 5.4. Clinical Monitoring and Management

Anti-amyloid mAbs require a recent baseline brain MRI and scheduled surveillance MRIs early in treatment to detect ARIAs promptly [83,135,136]. Because most ARIA events occur during titration, aducanumab monitoring is recommended before the 5th, 7th, 9th, and 12th infusions to maximize early detection [136]. For lecanemab, appropriate use recommendations advise MRI before the 5th, 7th, and 14th infusions, with an additional scan before the 26th infusion at week 52, especially in patients who carry APOE ε4 or patients with prior ARIA events [136]. Donanemab monitoring guidelines specify a baseline MRI within 12 months of treatment initiation, with exclusion of patients presenting >4 cerebral microbleeds, cortical superficial siderosis, or major vascular pathology [83]. Surveillance MRIs are recommended before the 2nd, 3rd, 4th, and 7th infusions, with an additional scan before the 12th infusion in higher-risk individuals, and at any point an ARIA is suspected clinically. Treatment discontinuation may be considered once amyloid clearance is documented by amyloid PET, typically at 12–18 months after initiation [83].

Management of ARIAs is guided by the severity of the event: mild, asymptomatic ARIAs, for example, small edema or a few microhemorrhages on MRI, may not require interrupting treatment; the patient can continue therapy with close monitoring and monthly MRI until lesions resolve [137]. However, any symptomatic ARIA or moderate-to-severe radiographic ARIA requires pausing therapy, with monthly MRI and supportive care until the ARIA changes resolve and symptoms fully resolve [138]. Severe ARIAs, such as extensive edema or any macrohemorrhage or recurrent ARIA episodes, may result in rapid permanent discontinuation of the antibody [138]. If dosing was suspended, treatment can be cautiously resumed after the ARIA has fully resolved on imaging and clinically, based on a careful risk–benefit reassessment and informed patient consent [136].

### 5.5. Global Regulatory Perspective

The regulatory approach of anti-amyloid mAbs has varied globally, reflecting different interpretations of evidence, safety priorities, and cost-value thresholds [139,140]. The United States FDA has taken a less restrictive approach, granting approval to three anti-Aβ antibodies, whereas the European Medicines Agency (EMA) and other agencies have been more cautious [139,140,141].

Aducanumab’s case demonstrated this difference: the FDA granted accelerated approval based on amyloid plaque reduction, despite debate over clinical benefit, whereas the EMA, Health Canada, and the UK Medical and Healthcare products Regulatory Agencies (MHRA) refused approval, citing insufficient efficacy data and safety concerns [142,143]. This decision provoked substantial controversy, including the resignation of three FDA advisory panel members and subsequent coverage restrictions by U.S. Medicare [65].

The EMA’s more conservative approach emphasizes that without clear functional improvement, risks such as ARIAs may outweigh potential benefit at a population level [62]. Even after additional confirmatory data that support lecanemab, the EMA restricted its approval to a narrower patient population, underscoring a cautious, precision-oriented implementation [144,145,146]. In contrast, donanemab, despite demonstrating similar clinical efficacy to lecanemab in TRAILBLAZER-ALZ 2 (~35% slowing on iADRS), was denied EMA authorization in early 2025 because of the persistent safety concerns, limited long-term data, and cost-effectiveness considerations [147].

These regulatory discrepancies highlight two contrasting philosophies: one prioritizing earlier access to promising therapies (FDA) and the other requiring more definitive evidence of clinical benefit and population-level value (EMA). Cost-effectiveness remains central to these decisions, with independent analyses (ICER) suggesting that current mAb prices far exceed value-based thresholds [139]. Such differences have wide implications for global equity and research investment, as delayed or limited access in Europe may affect trial participation and real-world evidence generation. Ongoing data from open-label extensions and registry-based monitoring may eventually harmonize global regulatory positions and clarify which patient subgroups derive the most meaningful benefit.

## 6. Biomarkers and Predictors of Response

Optimizing patient selection for these therapies relies on biomarkers that define AD pathology and stage. Amyloid biomarkers are a prerequisite: only patients with confirmed brain Aβ accumulation (positive amyloid PET or CSF Aβ42/tau ratio consistent with AD) should be treated [148,149,150]. This ensures the therapy targets the appropriate pathology; giving an amyloid-clearing drug to someone without amyloid (or with non-AD dementia) would only expose them to risk with no chance of benefit. In clinical trials, nearly all enrollees had biomarker-confirmed amyloid. In future practice, emerging plasma assays (for Aβ or phospho-tau) may serve as inexpensive screening tools, but confirmation with PET or CSF is recommended before initiating these costly and risk-bearing therapies.

### 6.1. Tau Pathology

The other hallmark of AD appears to modulate treatment benefit. High tau levels indicate advanced NFT spread, which correlates with more irreversible neuronal injury [151]. Post-hoc analyses and trial designs suggest patients with very high tau burden derive less benefit from amyloid clearance [150]. For example, donanemab’s Phase 3 trial enrolled only “intermediate” tau cases selected by PET, and an exploratory high-tau subgroup did not experience a slowing of progression [152,153,154]. This indicates that amyloid-directed antibodies are most effective in early stages before extensive tau-mediated neurodegeneration occurs, which is consistent with the amyloid cascade hypothesis, where amyloid triggers downstream tau pathology. Therefore, an optimal candidate might be in the mild stage with evidence of amyloid and only moderate tau burden. Tau PET scanning or CSF tau assays could, in the future, help stratify patients; for those with advanced tau for example in the medial temporal lobe and cortex, Braak stage V/VI might be better suited for clinical trials of anti-tau drugs rather than amyloid mAbs. Furthermore, both lecanemab and donanemab were shown to slow the increase in phospho-tau biomarkers as compared to the placebo group [29,76,150], indicating that effective amyloid clearance favorably alters the downstream tau pathology trajectory. Reductions in plasma p-tau_217_ and GFAP in donanemab-treated patients correlated with the degree of Aβ clearance [155,156], reinforcing that these drugs engage the disease biology. These fluid biomarkers might eventually be used longitudinally to gauge whether a patient is responding. However, this is an area of active research and not yet standard practice.

### 6.2. Genetic Factors

While APOE ε4 mainly affects risk and ARIAs, the degree of cognitive benefit is not clearly understood [67,157]. Other risk genes, such as TREM2, SORL1, and ABCA7, might influence an individual’s response, particularly TREM2, which is involved in microglial activation [158]. A rare TREM2 variant that impairs microglial Aβ clearance could hypothetically blunt the efficacy of antibody therapy, or alter ARIA risk, though this remains speculative [29]. As part of personalized care, detailed family history and even genetic testing for familial AD mutations (APP/PSEN1/PSEN2) may be appropriate in early-onset cases; patients with autosomal-dominant AD mutations will invariably develop amyloid pathology, and trials are ongoing to see whether treating such individuals pre-symptomatically with anti-amyloid antibodies can delay onset. In the coming years, polygenic risk scores and other biomarkers might refine patient selection further by identifying those likely to progress rapidly versus those with indolent diseases.

### 6.3. Precision Patient Selection, Cost, and Access Considerations

Precision patient selection and access considerations are crucial for translating anti-amyloid mAbs into real-world AD care. Treatment candidacy is generally limited to patients with biomarker-confirmed amyloid pathology (via PET imaging or CSF assays) and screening for APOE ε4 genotype and baseline MRI for cerebral microhemorrhages to alleviate the ARIA risk [80]. These meticulous protocols and metrics improve safety but require costly infrastructure, including amyloid PET, genotyping, and repeated MRI scans, which can limit access.

Financial barriers further complicate adoption: annual drug costs average ≈USD 26,500 (for lecanemab), and even with Medicare coverage, patients often face 20% coinsurance, adding roughly USD 300 per infusion if deductibles are unmet. For uninsured or underinsured individuals, out-of-pocket costs may exceed USD 1500 per infusion, including administration fees [159]. Coverage remains inconsistent, CMS provides conditional coverage through registries, but several private insurers require prior authorization or classify treatments as investigational, creating delays or denials [83,160]. These hurdles, along with the need for infusion center availability and repeated imaging, disproportionately affect rural and underserved populations, amplifying existing health disparities [83]. Clinically and from a policy perspective, combining precision selection with initiatives to expand reimbursement, subsidize biomarker testing, and improve infusion and imaging capacity is crucial for equitable implementation and widespread benefit.

## 7. Conclusions

Anti-amyloid mAbs have reshaped the therapeutic landscape of AD, representing the first therapies approved with disease-modifying intent. Aducanumab, lecanemab, and donanemab reduce amyloid plaque burden and, in early symptomatic AD, produce modest slowing of clinical decline. Their use, however, is constrained by heterogeneous efficacy and risks such as ARIAs, necessitating MRI monitoring and risk stratification, particularly by APOE ε4 status. A biomarker-guided strategy, requiring amyloid confirmation (PET or CSF), assessing tau burden, and incorporating plasma biomarkers (p-tau217, GFAP) and genetics, can improve patient selection and inform dose interruptions or discontinuation. The most significant net benefit is likely when therapy begins early, before extensive tau-mediated neurodegeneration. Priorities now include refining predictors of response and ARIA risk, establishing rules after plaque clearance, generating real-world effectiveness and safety data, and testing rational combinations (anti-tau, anti-inflammatory, and BBB-targeting agents) within multimodal care pathways. Taken together, anti-amyloid mAbs mark an essential first step toward precision therapy in AD; realizing their full impact will require better selection, safer delivery, and integration with complementary disease-modifying strategies.

## Data Availability

No new data were created or analyzed in this study. Data sharing is not applicable to this article.

## Abbreviations

ABCA7	ATP-binding cassette sub-family A member 7
AD	Alzheimer’s disease
ADCOMS	Alzheimer’s Disease Composite Score
ADAS-Cog14	Alzheimer’s Disease Assessment Scale–Cognitive subscale (14 items)
ADCS-ADL-MCI	Alzheimer’s Disease Cooperative Study–Activities of Daily Living for mild cognitive impairment
Aβ	amyloid-beta
APOE	apolipoprotein E
APP	amyloid precursor protein
ARIAs	amyloid-related imaging abnormalities
ARIA-E	ARIA with edema/effusion
ARIA-H	ARIA with cerebral microhemorrhages/siderosis
BBB	blood–brain barrier
CAA	cerebral amyloid angiopathy
CDR-SB	Clinical Dementia Rating–Sum of Boxes
CSF	cerebrospinal fluid
FDA	Food and Drug Administration
GFAP	glial fibrillary acidic protein
iADRS	Integrated Alzheimer’s Disease Rating Scale
MMSE	mini-mental state examination
MRI	magnetic resonance imaging
NFT	neurofibrillary tangle
NIA-AA ATN framework	National Institute on Aging–Alzheimer’s Association amyloid/tau/neurodegeneration framework
PET	positron emission tomography
p-Tau	phosphorylated tau
PSEN1	presenilin 1
PSEN2	presenilin 2
SORL1	sortilin-related receptor
Tau	microtubule-associated protein tau
TREM2	triggering receptor expressed on myeloid cells 2
